# Microwave-Assisted Plastic Upcycling: Dynamic Data Reconciliation, Parameter Estimation, and Kinetic Modeling

**DOI:** 10.1021/acs.iecr.6c01330

**Published:** 2026-07-02

**Authors:** Harish Damahe, Md Emdadul Haque, Chunlin Luo, Yuxin Wang, Jianli Hu, Debangsu Bhattacharyya

**Affiliations:** Department of Chemical and Biomedical Engineering, 5631 West Virginia University, Morgantown, West Virginia 26506, United States

## Abstract

Microwave (MW)-assisted catalytic pyrolysis offers a promising pathway for efficient plastic upcycling. This work develops an integrated modeling framework combining dynamic data reconciliation, a temperature-dependent rate model, and a yield model to represent the time-varying production rate of components in MW-assisted LDPE pyrolysis conducted in a batch reactor. An Arrhenius-type rate model with a temperature-dependent reaction order is developed. A biexponential correlation is proposed for the yield of gaseous products that enables to capture the evolving product formation behavior during conversion. In the yield correlation, one term is used to represent the initial increase in yield, reflecting the rapid formation of intermediate or primary products at the early stages of the reaction when a larger fraction of the reactant remains available. As conversion progresses, the influence of this term gradually diminishes. The other term accounts for the subsequent decrease in the predicted yield, representing secondary reactions such as further cracking or coke formation that reduce the concentration of certain products at higher conversion. The model is found to accurately represent reconciled experimental flow rate profiles from an in-house MW-assisted catalytic batch reactor for major products, including ethylene, ethane, 1-butene, and benzene, across 250–350 °C. Ethylene remains the dominant product but decreases from about 41.95% at 250 °C to 30.14% at 350 °C, while heavier products increase significantly, with 1-butene rising to nearly 8.37% and benzene reaching 2.17% at intermediate temperatures. The model shows that the ethylene production rate can be maximized at around 270 °C. The models developed in this work can be utilized for process optimization, reactor design and scale-up of microwave-assisted plastic conversion technologies, and economic analysis.

## Graphical abstract

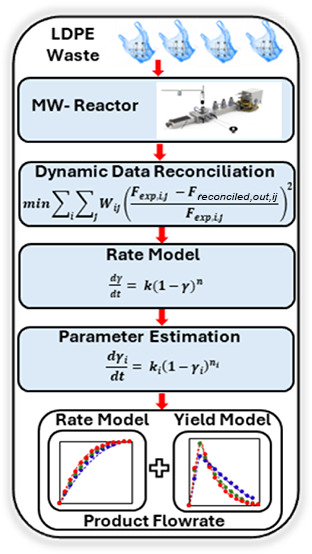

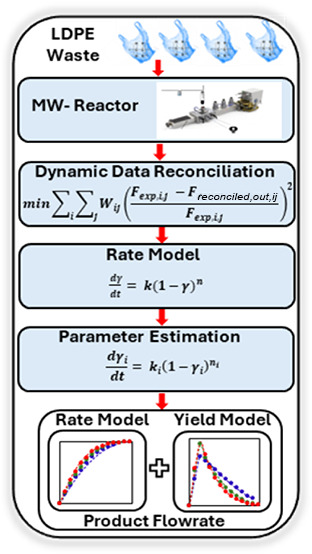

## 1 Introduction

In recent years, greenhouse gas (GHG) emissions have been an area of concern. The worldwide average composition of greenhouse gases in the environment is about 11.1% CH_4_, 6.1% N_2_O, 3.1% combined HFCs, PFCs, SF_6_, and NF_3_, and 79.7% CO_2_. In 2022, CO_2_ emissions reached a record high of 35.7 billion metric tons per year, and it is anticipated to rise to about 41.0 billion metric tons by 2050. The use of fossil fuels such as coal, natural gas, and petroleum oil plays a key role in the increase in CO_2_ emissions. The use of fossil fuels for power generation is being reduced with the increasing use of alternative energy sources such as solar, wind, geothermal, and biomass. One key use of petroleum oil is the production of petrochemicals including plastics, a large amount of which eventually ends up in landfills. As plastics are accumulating in the environment,^1^ it is highly desirable that they be recycled or upcycled.^2–4^ LDPE is the most abundant plastic found in municipal solid waste in the US. About 88% of LDPE goes to landfills, 10% is combusted, and only 2% is recycled. If ethylene, the monomer used for the production of LDPE, can be produced from LDPE, it would not only address the issue of accumulating LDPE in landfills but also lead to a circular pathway and reduce the use of current fossil-fuel-based processes for the production of ethylene, including ethane, propane, and naphtha cracking. A technology that is efficient and selective for ethylene production from LDPE can thus greatly reduce the environmental footprint of ethylene production, which is a leading source for CO_2_ emission. While technologies such as thermal pyrolysis and catalytic cracking processes have been used for recycling different types of plastics,^5^ these technologies are inadequate for the upcycling of LDPE since they produce small or negligible quantities of ethylene with numerous other undesired products such as aldehydes, ketones, and phenols.^6^

Various technologies are available for the recycling/upcycling of LDPE including chemical upcycling, biological upcycling, and mechanical upcycling.^5^ Biological upcycling uses certain types of bacteria, fungi, or enzymes to break down the plastic polymers.^7^ In mechanical recycling, cleaned plastics are shredded into small sizes, melted, and remolded to produce the desired product.^8^ Chemical upcycling can be done by converting the plastics through a catalytic or noncatalytic route such as pyrolysis, solvolysis, hydrogenolysis, gasification^9,10^ or functionalization (modifying the surface or chains).^9,10^ Thermal and thermo-catalytic pyrolysis has been used to convert different types of plastics including LDPE.^11–16^ However, the products are generally pyrolysis oil and gas without much selectivity.^13–15,17^ The pyrolysis reactions are often run at high temperatures reaching as high as 900 °C. MW-assisted noncatalytic and catalytic pyrolysis of LDPE has also been reported.^18–20^ MW-catalytic pyrolysis of LDPE can help to reduce the temperature considerably (as low as 200–250 °C) and be highly selective for the production of ethylene.^21,22^ MW-assisted catalytic conversion of LDPE can have a gas yield of 90 wt % at 300 °C, with about 23 wt % aromatics and 30 wt % light olefins.^22^

Kinetic modeling of pyrolysis processes can be broadly classified into model-free methods, model-fitting methods, and both.^15^ Model-free methods include the Friedman method (FR),^23^ Kissinger–Akahira–Sunose method,^24,25^ Flynn–Wall–Ozawa method,^26,27^ Coats–Redfern method,^28^ Criado method,^29^ Starink method,^30^ and Distributed activation energy model (DAEM).^31^ These models consider that the reaction rates change only with temperature.^23–32^ Model-fitting methods include Reaction-order models, Geometrical contraction/phase boundary models, Diffusion models, Nucleation and growth models, Power law models,^33–35^ and variants of typical Arrhenius-type models.^36^ Besides these, some methods such as Coats–Redfern method,^28^ Prout–Tompkins model,^37^ Freeman–Carroll method,^38^ Horowitz–Metzger method,^39^ and Kissinger methods share principles from both model-free and model-fitting methods. These models have been applied to thermal and thermo-catalytic degradation processes.^6,40–42^ Kinetic models of LDPE thermal pyrolysis have been developed using TGA data through model-free and model-fitting methods,^43^ using temperature and heating rate (β) as inputs to compute the remaining mass fraction at any time instant.^43^ For MW pyrolysis of LDPE, a quadratic model has been developed for estimating oil productivity (wt %) as a function of pyrolysis temperature, N_2_ flow rate, and the catalyst (SiC)-to-LDPE ratio.^18^ In both model-free and model-fitting methods referenced earlier, pyrolysis reaction rates are given as a function of temperature and fractional conversion. The dependence on reaction rate is generally given by an Arrhenius-type relation, but the dependence on fractional conversion rate can greatly vary depending on whether a model-free or model-fitting method is used and the assumed mechanism and reaction order (such as exponential nucleation, random nucleation and nuclei growth, phase boundary-controlled reactions, diffusion-limited reactions, and their variants). For MW-assisted catalytic pyrolysis of LDPE, it has been observed that it is not only the reaction rate that is a function of temperature and fractional conversion, but the yields of all products are also highly nonlinear functions of temperature and fractional conversion (or remaining mass or time for a batch pyrolysis reactor).^22^ None of the kinetic models referenced earlier can be used to represent the variation in yield as a function of temperature and fractional conversion in the experimental data.^22^ While kinetic models for similar variability in yield and reaction rate for MW-assisted chemical reactions, such as methane dehydroaromatization (DHA), have been presented earlier by some of the authors of this paper,^44,45^ in those studies, the reason for the time-varying change was coke formation. Therefore, a model for coke formation and accounting for the accumulation of coke was used to represent the variability in yield and conversion. However, for MW-assisted catalytic pyrolysis, the variability in yield is not due to coke formation. In addition, the yield for MW-assisted catalytic pyrolysis follows highly complex dynamic characteristics.^22^ To the best of our knowledge, a kinetic model that can represent the highly nonlinear variability in reaction rate and yield as a function of time and fractional conversion for MW-assisted catalytic pyrolysis of LDPE is not currently available in the existing literature. In what follows, as the data for kinetic model development are obtained from a batch reactor, we interchangeably refer to the variability in yield as time-varying yield.

In this study, a kinetic model is developed for the upcycling of LDPE in an MW-assisted pyrolysis process. It is expected that the carbon-to-hydrogen ratio of 0.5 on a molar basis must be satisfied by the experimental data, not necessarily by the instantaneous product flow rates, but if the carbon and hydrogen contained in the cumulative amount of all products over the entire batch time are considered. However, it was observed that the experimental data did not satisfy this constraint. Therefore, a dynamic data reconciliation (DDR) problem is solved for reconciling the experimental data to be used for kinetic model development. For obtaining the cumulative amount of all products during the batch time, it is desired that the experimental data be available continuously. However, this is not feasible. The in-house experimental data for product flow rate used in this work are only available every 3 min. A numerical integration technique is applied with the trapezoidal rule to obtain the integral carbon and hydrogen produced during the entire duration of the experiment while performing the DDR. A kinetic model for time-varying conversion is developed, and its parameters are estimated using the in-house experimental data. In addition, yield models are proposed, and their parameters are optimally estimated for representing the time-varying yield of each product as a function of temperature. Finally, the yield model is coupled with the kinetic model to represent the transient production rate of all products as a function of the target temperature.

The main contributions of this paper are as follows:

- Performed dynamic data reconciliation for the MW-assisted catalytic pyrolysis of LDPE at three operating temperatures.
- A reaction rate model was developed and its parameters were estimated using the experimental data.
- Yield models for products were developed and their parameters were optimally estimated using the reconciled experimental data.
- The model-estimated production rate was validated with the experimental data.
- Studied sensitivities of time-averaged yield with respect to temperature.

This paper is organized as follows: Section 2 presents the experimental procedure. Section 3 presents the methodology of this study. Section 4 presents the results and discussion. Finally, Section 5 presents the conclusions and future work.

## 2 Experimental Procedure

### 2.1 Chemicals and Catalyst Preparation

LDPE films received from the real world were utilized in the experiment. Materials for the catalyst, including α-Fe_2_O_3_ and ruthenium­(III) chloride (47.7% Ru content, 3.110 g/mL), were purchased from Sigma-Aldrich. The Ru/α-Fe_2_O_3_ catalyst (4 wt % Ru) was synthesized via a wet impregnation technique.^46^ Ruthenium­(III) chloride was dissolved in 50 mL of deionized water and ultrasonicated for 10 min. Then, 5 g of α-Fe_2_O_3_ was added to the solution, and the mixture was stirred at 400 rpm overnight. The resulting mixture was dried at 100 °C for 6 h and then calcined at 550 °C for 4 h. Finally, the catalyst was reduced under pure H_2_ at 150 °C for 6 h with a heating rate of 2 °C/min. The details of catalyst preparation can be found in a previous publication by some of the authors.^46^

### 2.2 Experimental Setup and Procedure

Figure 1 shows the schematic of the experimental setup for the MW-assisted catalytic process. The MW-assisted catalytic system was powered by a Sairam microwave control system, operating with a 2.45 GHz solid-state generator and a maximum forward power of 1 kW. A TE10 monomode cavity served as the applicator, ensuring ideal electric conductivity. An infrared pyrometer monitored the reaction temperature, adjusting the microwave power accordingly. The controlled variables for this system, namely the reaction temperature, ramp rate, and time-on-stream, were regulated by a CBA Eurotherm controller. A crystal detector measured the reflected power, and both the forward and reflected power were logged throughout the upcycling process. The initial dwell segment lasted for approximately 1 min at a temperature of 160 °C, during which autotuning was employed. A heating rate of 20 °C/min was used by adjusting the forward power. Once the target temperature was reached, the microwave power decreased and stabilized for the 40 min hold period. The external surface temperature was measured using an infrared thermometer, and thermal images were captured by a FLIR infrared camera to track the temperature distribution and changes during the reaction.

**Figure 1:**
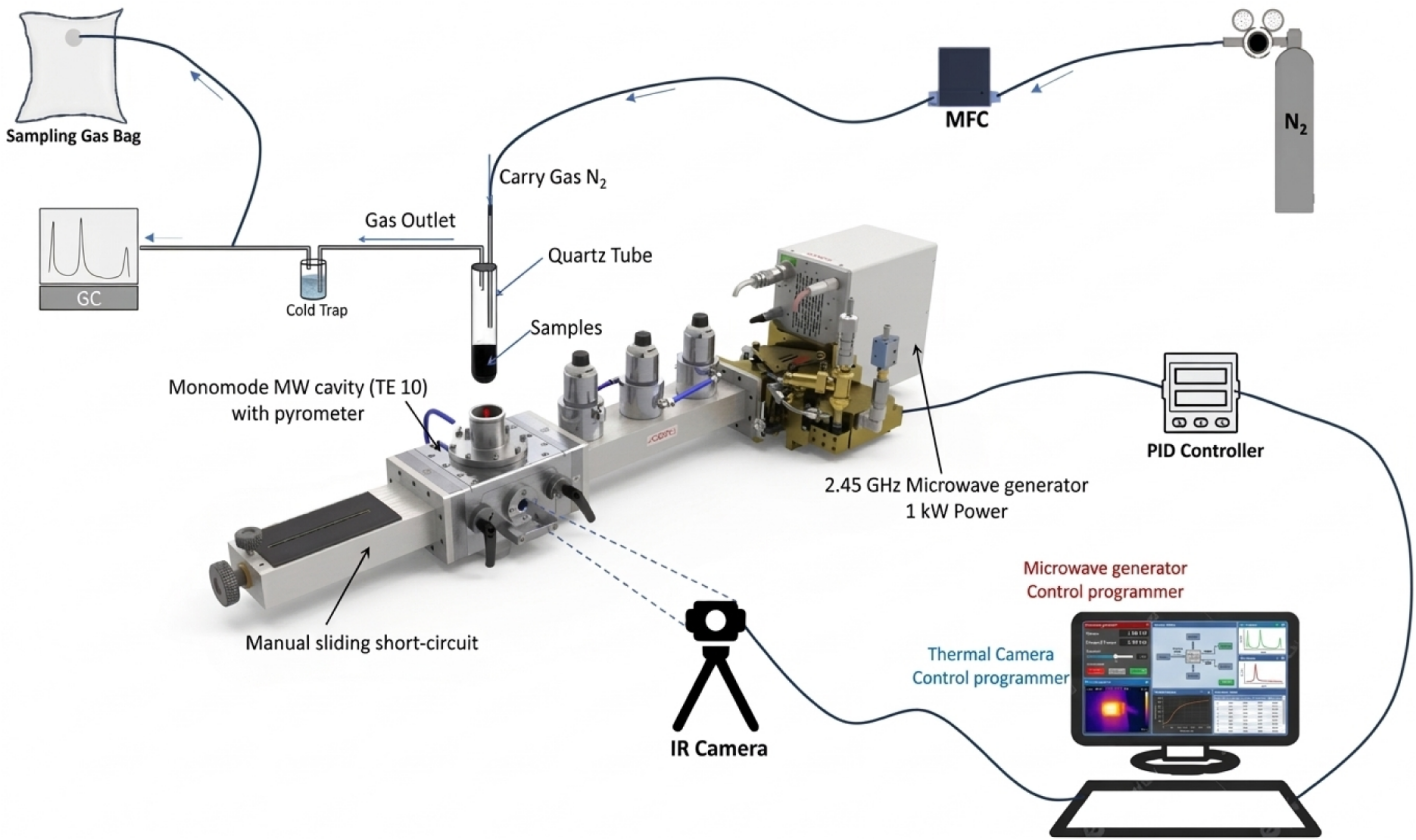
Schematic diagram of the MW-assisted catalytic upcycling setup.

Product concentrations were measured using a four-channel INFICON Fusion Micro-GC. The instrument was equipped with an Rt-Molsieve 5A column for O_2_, N_2_, H_2_, CO, and CH_4_; an Rt-V-Bond column for CO_2_, C_2_H_6_, C_2_H_4_, and C_2_H_2_; an Rt-Alumina Na_2_SO_4_ column for C_3_–C_4_ hydrocarbons and olefins, including propane, propylene, 1-butene, and isobutylene; and an Rxi-1 ms column for aromatic products such as benzene and toluene. Based on the information available in the Micro-GC manufacturer’s manual, the μTCD can achieve detection limits down to approximately 1 ppm for selected compounds under optimized methods, although the actual sensitivity depends on the compound, carrier gas, and sample matrix.

We would like to clarify that although benzene and toluene are liquids at ambient temperature, they can be partially transported in the vapor phase by the continuous N_2_ carrier gas. To avoid condensation, the outlet transfer line was maintained at approximately 120 °C, which is above the boiling points of benzene and toluene, to ensure that benzene and toluene purged from the reactor could pass through the heated line and be detected by the Micro-GC.

The in-house experimental data were obtained from different experiments undertaken at each target temperature. For a given target temperature, each reaction condition was independently repeated three times, and the reported data that were used for model development represent the average values from triplicate experiments. Additional information about the experimental method can be found in a previous publication by some of the authors.^46^

## 3 Methodology

### 3.1 Dynamic Data Reconciliation

A data reconciliation technique is applied to the in-house experimental data collected for three different operating temperatures: 250 °C, 300 °C, and 350 °C. A gas chromatograph is connected to the reactor outlet to measure the gas composition over time. Experimental data are dynamic, i.e., time-on-stream data, that are collected at 3 min interval. Due to measurement noise and sensor uncertainty, the experimental data do not necessarily satisfy the carbon-to-hydrogen balance. To address these inconsistencies and ensure elemental balance, an optimization-based data reconciliation problem is formulated to minimize the deviation between experimental data and model-predicted values. The DDR formulation is presented in eq 1. Inequality constraints defined in eq 2 ensure that the molar flow rates remain positive, whereas eq 3 enforces a carbon-to-hydrogen ratio of 0.5 by considering the number of carbon and hydrogen atoms contained in the cumulative number of moles of all products during the entire duration of the experiment. It should be noted that based on the closure of mass balance (i.e., mass of feed vs mass of all products that are accounted for), unmeasured nonvolatile products, such as coke and heavy hydrocarbons, are expected to be negligible for these specific MW-assisted catalytic reactions that occur at much lower temperatures compared to conventional thermal pyrolysis of LDPE. The cumulative number of moles of each product is calculated by numerical integration of the reconciled flow rate by using the trapezoidal rule since the measurements are only available at discrete intervals but the carbon-to-hydrogen ratio needs to be satisfied by considering the intermediate values, for which measurements are not available. The optimization problem is solved by using MATLAB’s “Fmincon” function, using the sequential quadratic programming (SQP) algorithm.

1 $$\min\underset{i}{\sum }\underset{j}{\;\sum }{\;W}_{ij}{\;(\frac{F_{\exp,i,j}-F_{reconciled,out,i,j}}{F_{\exp,i,j}})}^{2}$$

*s.t.*

2 $$F_{reconciled,out,i,j}\geq 0$$

3 $$C_{cumulative}/H_{cumulative}=0.5$$

In eqs 1–3, index *i* refers to the gaseous species, while index *j* signifies each discrete time instant at which measurements are recorded.

### 3.2 Reaction Rate Model

MW-assisted catalytic pyrolysis of LDPE yields an ethylene product as a monomer. It also yields several other products.^36^ The reactor temperature is substantially lower for the MW-assisted process (often in the range of 250–350 °C) compared to energy-intensive conventional methods such as thermal degradation and thermal catalytic processes. The products of the MW-assisted pyrolysis process are predominantly gaseous,^22^ with the gaseous products mainly including light olefins^22,47^ as opposed to the thermal degradation of LDPE that dominantly produces liquid, gas, and solid.

The reaction rate model considered for the reactions of LDPE pyrolysis is given by eq 4–7. Eq 4 denotes the dimensionless weight fraction of the LDPE, where γ ranges from 0 to 1. The kinetic rate model given by eq 5 describes the temporal evolution of the normalized conversion (dimensionless weight fraction) γ, where *k* is the rate constant and *n* is the reaction order, while (1 – γ) denotes the unconverted fraction of the LDPE. As the reaction progresses and γ approaches unity, the term (1 – *γ*)^*n*^ decreases, leading to a gradual reduction in reaction rate. The parameter *n* controls the kinetic behavior: *n* = 1 corresponds to a first-order decay model, whereas *n* ≠1 introduces nonlinearity that can help to represent complex reaction mechanisms. In this work, the reaction order *n* is expressed as a temperature-dependent parameter using the correlation given by eq 6, where *T* is the reaction temperature and *a*, *b*, and *c* are empirical coefficients that are optimally estimated. It should be noted that *n* represents an apparent reaction order rather than a fundamental mechanistic quantity. We have observed that a constant reaction order leads to high error in the model results in comparison to the experimentally observed conversion rate over the investigated temperature range. Therefore, the temperature dependence of *n* is introduced to represent the observed complex temperature dependence of the conversion rate at a given fractional conversion. While the authors are currently not able to provide a mechanistic explanation of the observed temperature dependence of the reaction order, we believe that this stems forth due to expected complex reaction pathways involved in MW-assisted catalytic LDPE pyrolysis that are likely to include multiple parallel and consecutive reactions, including depolymerization reactions, secondary cracking, and catalytic transformation. The rate constant *k* is given by the Arrhenius equation, which is expressed in eq 7, where *A* is the pre-exponential factor, *E*_a_ is the activation energy, *R* is the gas constant, and *T* is the reaction temperature.

4 $$\gamma =\frac{w_{i}-w_{t}}{w_{i}-w_{f}}$$

5 $$\frac{d\gamma }{dt}=k{\left(1-\gamma \right)}^{n}$$

6 $$n=aT^{2}+bT+c$$

7 $$k=Ae^{-E_{a}/RT}$$

The energy conservation equation given by eq 8 describes the transient temperature behavior of the reactor by considering heat supplied to the system, the enthalpy changes due to chemical reactions, and heat exchange with the surroundings. The energy conservation equation is written as follows:

8 $$\frac{d}{dt}\left(\sum w_{i}Cp_{i}T\right)=Q_{MW}-\left(\Delta H_{rx}\right)\left(\frac{dw_{t}}{dt}\right)-{ṁ}_{gas,out}{Ĥ}_{gas}$$

where *w*_*i*_ is the mass (*i* = solid, gas), *Cp*_*i*_ is the specific heat, *T* is the temperature (assuming gas and solid temperatures are identical), Δ*H*_*rx*_ is the heat of the reaction, *Q*_*MW*_ is the heat input from MW-induced heating, ṁ_*gas,out*_ is the mass flow rate of outgoing gaseous products, and Ĥ_*gas*_ is the specific enthalpy of gaseous product. The initial condition for the dynamic model given by eqs 4 and 5 is when the reactor temperature is 160 °C. By using the given temperature profile of the MW reactor, eq 8 is used to estimate time-varying *Q*_*MW*_ that can be utilized for process modeling and economic analysis.

### 3.3 Parameter Estimation for Reaction Rate Model

A dynamic parameter estimation problem for the proposed kinetic model is performed. The parameter estimation problem is formulated as an optimization problem given by eqs 9–13. The objective function represented by eq 9 is to minimize the discrepancy between reconciled experimental data and model predictions. Inequality constraints given by eq 10 ensure that the cumulative conversions remain positive. Eqs 11–13 represent the kinetic model. Decision variables are the activation energy (*E*), the pre-exponential factor (*A*), and coefficients (*a*, *b*, *c*) for the reaction order *n*.

9 $$\min\underset{i}{\sum }\underset{j}{\sum }{\left(Cumuconv_{\exp,i,j}-Cumuconv_{\mathrm{mod}el,i,j}\right)}^{2}$$

s.t.

10 $$Cumuconv_{model,i,j}\geq 0$$

11 $$\frac{d\gamma_{i}}{dt}=k_{i}{\left(1-\gamma_{i}\right)}^{n_{i}}$$

12 $$n_{i}=a{T_{i}}^{2}+bT_{i}+c$$

13 $$k_{i}=Ae^{-E/RT_{i}}$$

Here, index *i* denotes the specific operating temperature for which the experimental data are available, while index *j* denotes each discrete time point for which measurements are available. *Cumuconv*_*exp*_ and *Cumuconv*_*model*_ represent the cumulative conversion in the experimental data and from the model, respectively, at the corresponding temperature. The parameter estimation problem is solved using MATLAB’s “fmincon” function with the sequential quadratic programming (SQP) algorithm. Lower and upper bounds are imposed on the decision variables. Data for three temperatures (250 °C, 300 °C, and 350 °C) available from in-house experiments are used.

### 3.4 Yield Model

The yield model is developed to quantify the individual species as a function of the temperature and the remaining LDPE mass under time-varying conditions. The overall reaction of LDPE pyrolysis is presented in eq 14:

14 $${\left(C_{2}H_{4}\right)}_{n}\to \overset{N}{\underset{i=1}{\sum }}\alpha_{i}S_{i}$$

where index *i* represents the gaseous product and *N* represents the total number of product species. The gaseous products are denoted by *S*_*i*_ where *S*_*i*_ ∈ {*H*_2_,*CH*_4_,*C*_2_ *H*_4_,*C*_2_ *H*_6_,*C*_3_ *H*_6_,*C*_3_ *H*_8_,*C*_4_ *H*_8_,*C*_6_ *H*_6_,*C*_7_ *H*_8_}, and α_*i*_ is the respective yield coefficient of gaseous product *i*.

To determine the yield coefficient of gaseous products, a nonlinear biexponential correlation is developed and presented in eq 15. The proposed formulation consists of two exponential components that enable the yield coefficient to capture evolving product formation behavior during conversion. The first term represents the initial increase in yield, reflecting the rapid formation of intermediate or primary products at the early stages of the reaction when a larger fraction of the reactant remains available. As conversion progresses, the influence of this term gradually diminishes due to its exponential decay. In contrast, the second term accounts for the subsequent decrease in the predicted yield, representing secondary reactions such as further cracking or coke formation that reduce the concentration of certain products at higher conversion. The parameters *a*_*i*_, *b*_*i*_, *c*_*i*_, *d*_*i*_, and *e*_*i*_ in eq 15 are estimated using yield data obtained from the reconciled experimental results. Among these, *a*_*i*_, *d*_*i*_, and *e*_*i*_ are treated as parameters that do not depend on temperature, whereas *b*_*i*_ and *c*_*i*_ are considered to be temperature-dependent parameters represented by eqs 16 and 17. Parameters *b*_*i*_ and *c*_*i*_ are represented by temperature-dependent sigmoid functions as these functions are smooth across different reaction temperatures. Eq 16 represents *b*_*i*_ that includes a scaling parameter and a temperature-dependent sigmoid function added with a constant (i.e., independent of temperature). This helps to capture a characteristic that rises with temperature eventually yielding a constant value at higher temperatures. In contrast, eq 17 utilizes a composite bisigmoidal formulation consisting of two opposing logistic components, which provides additional flexibility to represent more complex temperature-dependent behavior, including growth and decay trends.

15 $$\alpha_{i,j,k}=a_{i}\cdot \left(1-d_{i}\cdot e^{\left(-b_{i}\cdot \left(1-W_{rem,j}\right)\right)}+e_{i}\cdot e^{\left(-c_{i}\cdot \left(1-W_{rem,j}\right)\right)}\right)$$

16 $$b_{i}=b_{sc,i}\cdot \left(\frac{1}{\left(1+e^{\left(-b_{1,i}\cdot \frac{T_{k}}{T_{sc}}-b_{2,i}\right)}\right)}+b_{3,i}\right)$$

17 $$c_{i}=c_{sc,i}\cdot \left(\frac{1}{\left(1+e^{\left(c_{1,i}\cdot \frac{T_{k}}{T_{sc}}-c_{2,i}\right)}\right)}+\frac{c_{3,i}}{\left(1+e^{\left(-c_{1,i}\cdot \frac{T_{k}}{T_{sc}}-c_{2,i}\right)}\right)}\right)$$

In eqs 15–17, *i* represents the gaseous species, *j* represents each discrete time point for which measurements are available, *k* denotes the specific operating temperature for which the experimental data are available, *T* is the reaction temperature, *Tc*_*sc*_ is the scaling factor (considered to be the highest temperature for which experimental data are available, i.e., 350 °C for this work), and *W*_*rem*_ is the mass remaining.

For parameter estimation, the following optimization problem is solved:

18 $$\min\underset{i}{\sum }\underset{j}{\sum }\underset{k}{\sum }{\left(\alpha_{\exp,i,j,k}-\alpha_{\mathrm{mod}el,i,j,k}\right)}^{2}$$

s.t.

19 $$0\leq \alpha_{model,i,j,k}\geq 1$$

20 $$\underset{i}{\sum }\alpha_{model,i}=1,\forall j,k$$

21 $$\alpha_{i,j,k}=f\left(T_{k},W_{rem,j}\right)$$

Eq 19 constrains that yields remain bounded between 0 and 1. Eq 20 constrains that the summation of yields at any time instant for all temperatures remains unity. Eq 21 denotes the yield model given by eqs 15–17. It should be noted that when a model like eq 15 is used for predicting the yield for any arbitrary condition, they may not sum up to 1. Therefore, the computed yield coefficients from eq 15 must satisfy eq 19 and must be normalized to ensure that they satisfy eq 20.

### 3.5 Computing Product Flow Rate

For a process simulation, it is desired that the product flow rates be computed. Product flow rates at any instant of time are computed by combining the results from the reaction rate model (given by eqs 4–7) and the yield model (given by eqs 15–17) at the same instant. It is desired that the product flow rates satisfy eq 3. To achieve that, the H_2_ yield is considered to be the degree of freedom. Then all product yields are normalized to ensure that they satisfy eq 20. These normalized yields and the reaction rate models are used to compute the final product flow rates that are then compared with the experimental data.

## 4 Results and Discussions

### 4.1 Reconciled Data

The average root-mean-square error (RMSE) (%) between the reconciled data and the experimental data for individual components obtained from DDR is listed in Table 1. Overall, the reconciled profiles closely follow the experimental data while removing unexpected variability and enforcing physically consistent trends. It can be observed in Table 1 that methane has the highest error, while isobutylene has the least error. The key target product for this MW-assisted conversion is ethylene, for which a very low error is observed. It should be noted that as expected ethylene is the most dominant component obtained from this process, whereas toluene is the least produced product across the entire temperature range that is investigated in this study.

**Table 1:** RMSE (%) for DDR

Species	Methane	Ethylene	Ethane	Propane	Propylene	1-Butene	Toluene	Iso-butylene	Benzene
RMSE	8.06	0.32	3.00	1.56	0.10	0.03	3.92	0.01	1.26

### 4.2 Reaction Rate Model Validation

The estimated parameters for the reaction rate of MW-assisted LDPE pyrolysis are summarized in Table 2. In Table 2, rate parameters obtained for the thermal degradation of LDPE are also included.^11^ It is interesting to note that the activation energy *E* is significantly lower (13 kJ/mol) for the MW-assisted process compared to thermal pyrolysis (177.20 kJ/mol), indicating that MW-assisted pyrolysis can have a similar reaction rate to thermal pyrolysis at a much lower temperature than thermal pyrolysis and therefore is likely to improve the thermal efficiency of LDPE pyrolysis.

**Table 2:** Estimated Kinetic Parameters and Comparison with Thermal Process

Parameter	Value (MW)	Value (Thermal)^11^
A	62.12 sec^–1^	1.10 × 10^14^ sec^–1^
E	13.0 $\frac{kJ}{mol}$	177.20 $\frac{kJ}{mol}$
a	1.85 × 10^–6^	N/A
b	5.28 × 10^–6^	N/A
c	2.06 × 10^–5^	N/A

Figure 2 shows the comparison between the model results and experimental data for cumulative conversion of LDPE at 250 °C, 300 °C, and 350 °C. For all temperatures, cumulative conversion increases rapidly during the initial reaction period and gradually approaches unity indicating complete consumption of LDPE at all temperatures. As temperature increases, the conversion rate increases as expected, thus reaching complete conversion earlier. The model results closely follow the reconciled data across the entire time range, with an average RMSE value of 2.95%..

**Figure 2:**
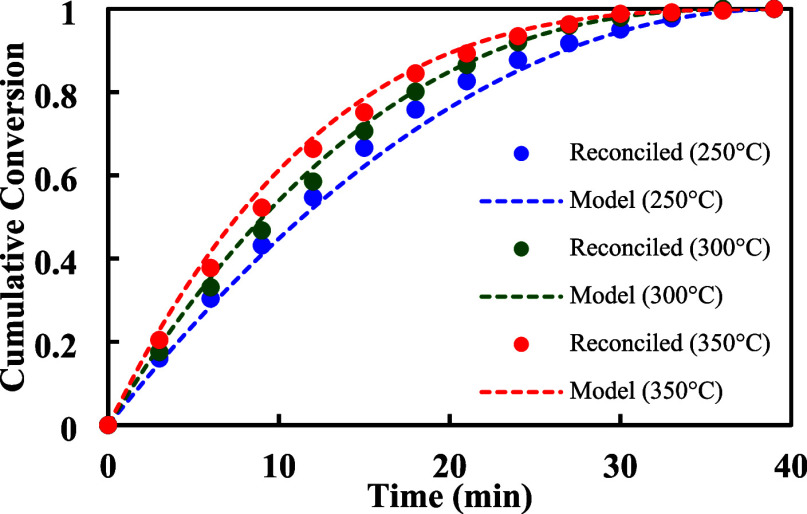
Comparison of model results with the experimental data for cumulative conversion.

### 4.3 Yield Model Validation

Figure 3 compares the reconciled yield coefficients with the corresponding model predictions for ethylene, ethane, 1-butene, and benzene at 250, 300, and 350 °C with variations in the remaining mass fraction. The estimated parameters for the yield model of each component are listed in Table S1.

**Figure 3:**
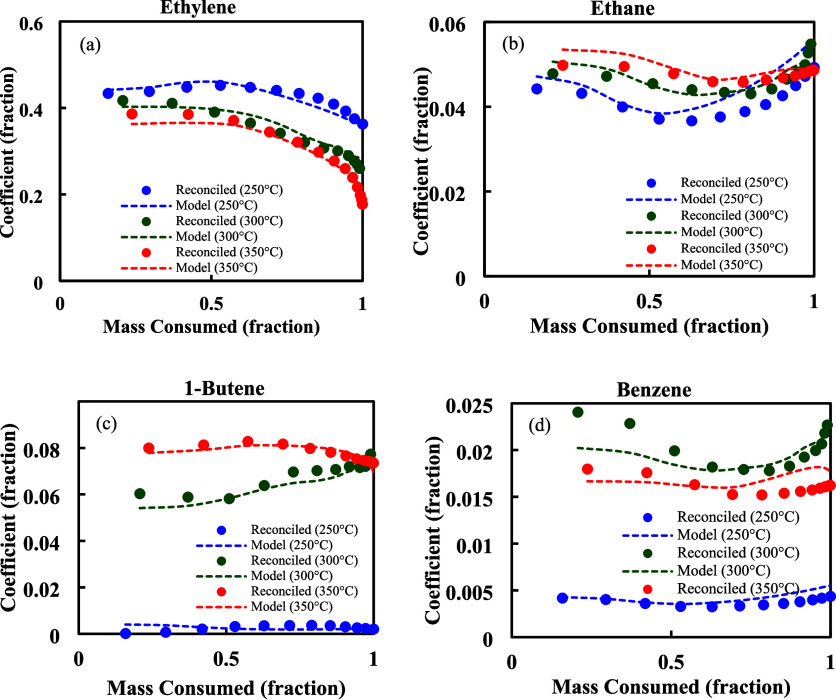
Comparison between reconciled data and model results for the yield of (a) ethylene, (b) ethane, (c) 1-butene, and (d) benzene at 250 °C, 300 °C, and 350 °C.

In Figure 3a, it can be observed that ethylene yield reasonably remains flat for all temperatures for most of the duration of the batch time and then decreases as the fraction of mass consumed increases (i.e., after some batch time has elapsed) beyond a certain value. Ethylene yield is also found to decrease with temperature irrespective of the fraction of mass consumed reaching peak values of approximately 0.46 at 250 °C, 0.42 at 300 °C, and 0.39 at 350 °C. The decrease in the ethylene yield fraction at high conversion is steeper at higher temperatures. This behavior indicates that ethylene likely degrades to form other products as the temperature is increased. On the contrary, ethane (see Figure 3b), 1-butene (see Figure 3c), and hydrogen (see Figure S1a) yields increase at higher temperatures indicating the likely products that are formed from the degradation of ethylene as the temperature is increased. Figure 3b shows that ethane yield exhibits high nonlinearity—it decreases with the increase in the fraction of mass consumed and then starts rising beyond a batch time (i.e., beyond a fraction of mass consumed). It can be observed in Figure 3b that the developed model can very accurately represent the complex, nonlinear time-varying yield of ethane.

As seen in Figure 3c, 1-butene demonstrates strong temperature dependence, with negligible yield at 250 °C, and significantly higher peak values at elevated temperatures, reaching approximately 0.073 at 300 °C and 0.083 at 350 °C as peak values. It can also be seen in Figure 3c that 1-butene yield decreases at 350 °C beyond a certain value of the fraction of mass consumed, but it increases at 300 °C with the increase in the fraction of mass consumed. It can be observed that the yield model could represent the complex characteristics of 1-butene yield with respect to temperature and the fraction of mass consumed very well. It is interesting to note in Figure 3 that while ethylene, ethane, and 1-butene show a monotonic trend with temperature (i.e., yield either increases or decreases with the increase in temperature), benzene yield does not show monotonicity with respect to temperature. Figure 3d shows that the benzene yield is very low at 250 °C, increasing by about 4 times as the temperature is raised to 300 °C. At 350 °C, the benzene yield drops compared to that at 300 °C. Variability in the benzene yield with respect to the fraction of mass consumed is similar to that of ethane yield. Figure 3d shows that the yield model can represent these highly nonlinear characteristics of benzene yield with temperature and mass consumed very well. Overall, the close agreement between reconciled experimental data and model predictions across all temperatures confirms that the combined biexponential yield formulation and sigmoidal temperature correlations provide a representative and consistent framework for describing the evolution of product yields during MW-assisted LDPE pyrolysis. The results for other species, such as hydrogen, methane, propylene, propane, isobutylene, and toluene, are provided in Figure S1 in Supporting Information. The corresponding average RMSE for yield calculated over the entire batch time and all three temperatures is listed in Table S2. While the average RMSE values for hydrogen, methane, ethylene, propylene, and propane are below 10%, the average RMSE values for benzene, toluene, and isobutylene are 11.95, 14.16, and 28.25%, respectively. One key reason for the high error is the very low yield values of these components. For example, when the experimental data for isobutylene corresponding to 250 °C (the corresponding average yield fraction is only 0.002) are excluded from the average RMSE calculation, the average RMSE becomes 8.5% (see Table S2). In addition, these errors are not likely to have much impact on process design or economic analysis as the flow rates of these components are significantly lower than those of ethylene, the key target product, and propylene, the other dominant product that is of interest. Cumulative production of ethylene during the entire batch time is typically found to be more than 200 times that of toluene and 60 times that of isobutylene at all temperatures that are investigated.

Finally, as mentioned earlier, we would like to note that for a parametrized, data-driven yield model such as the one developed in this work, for its implementation in a process model, the yield parameters should be computed using the yield model with the estimated parameters, but must be constrained by eqs 19 and 20.

### 4.4 Validation of Product Flow Rate

Figure 4 compares the reconciled flow rates of ethylene, ethane, 1-butene, and benzene at 250 °C, 300 °C, and 350 °C with their respective model results.

**Figure 4:**
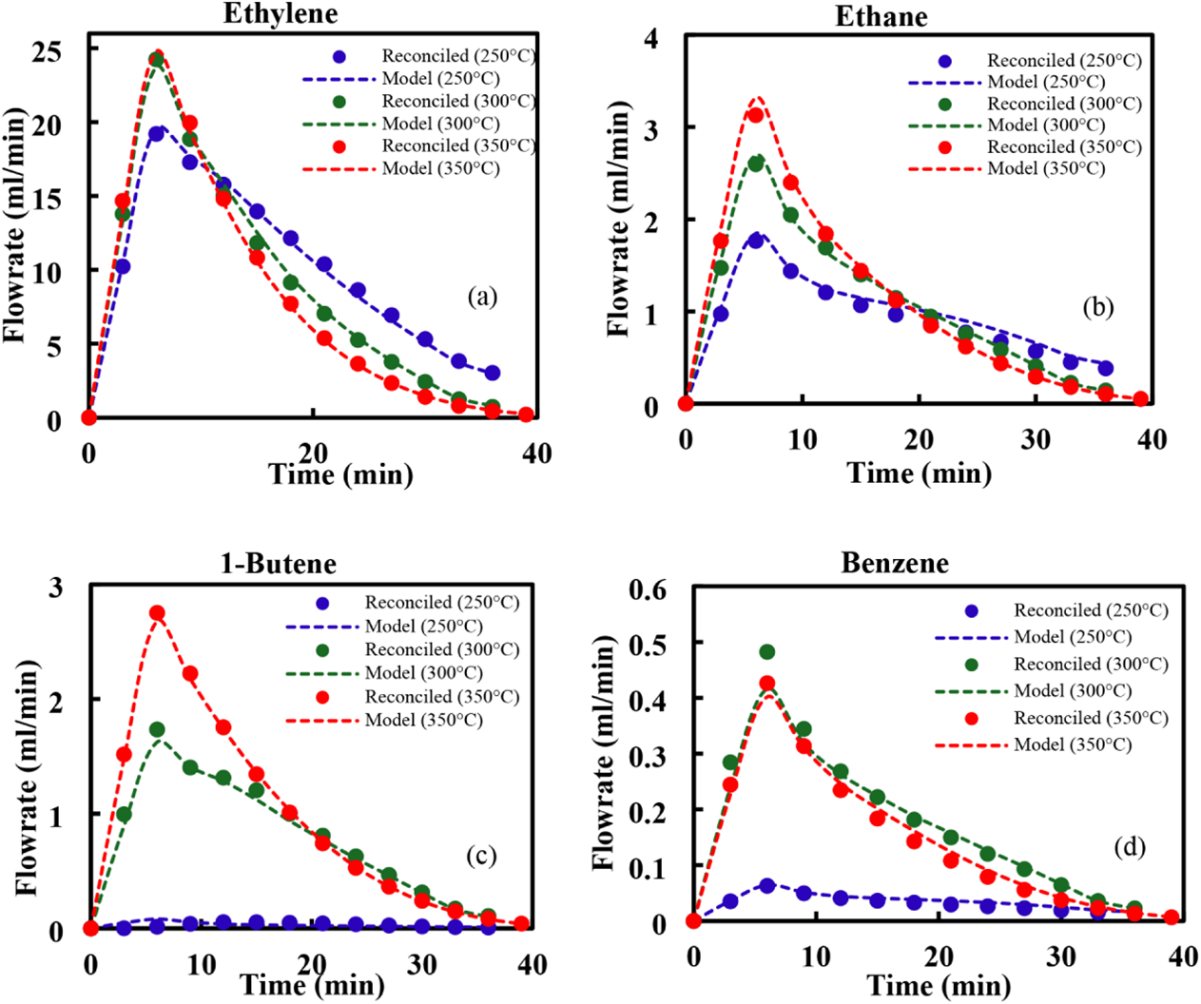
Comparison between reconciled data and model results for the flow rate of (a) ethylene, (b) ethane, (c) 1-butene, and (d) benzene at 250 °C, 300 °C, and 350 °C.

Ethylene remains the dominant product but shows decreasing selectivity with increasing temperature, with yields declining from approximately 41.95% at 250 °C to 37.86% at 300 °C and 30.14% at 350 °C. In contrast, ethane exhibits a modest increase, rising from about 4.18% at 250 °C to nearly 5.01% at 350 °C. Formation of heavier olefins becomes more pronounced at elevated temperatures. For example, 1-butene increases significantly from approximately 0.24% at 250 °C to 6.67% at 300 °C and 8.01% at 350 °C. Similarly, benzene formation increases from roughly 0.39% at 250 °C to about 2.07% at 300 °C, but then decreases to 1.7% at 350 °C. The results for remaining species such as hydrogen, methane, propylene, propane, isobutylene, and toluene are provided in Figure S2 in the Supporting Information. RMSE for the flow rate of individual elements is calculated by considering all measured flow rates of that component for each reactor temperature over the entire batch time. These values are listed in Table S2. For key products of interest, ethylene and propylene, RMSE is found to be below 10%. While RMSE for benzene, toluene, and isobutylene exceeds 10% (as would be expected based on the error from the yield model described earlier), the key reason for the high error is the very low flow rates of these components as noted earlier compared to dominant products such as ethylene and propylene. Overall, the combined kinetic-yield model is found not only to capture the dynamic flow rate behavior (i.e., change in the product flow rate with time or amount of mass consumed), but also to represent well the temperature-dependent product distribution.

### 4.5 Sensitivity to Temperature

Parameter estimation and model validation are conducted for three reaction temperatures at which experimental data are available—250, 300, and 350 °C. It is observed that an increase in temperature leads to faster conversion (i.e., shorter batch time); however, this does not necessarily imply a corresponding increase in product yield. It is also observed that there is high nonlinearity. The developed model is used to perform sensitivity analysis to temperature to observe what is expected to be the optimal temperature for ethylene yield, the key desired product from the process. The yield at any temperature is the time-averaged yield over the entire batch time.

Figure 5a shows the estimated yield (%) variation with temperature between 250 and 350 °C for ethylene and propylene. Figure 5b shows the results for benzene, toluene, and isobutylene. Figure 6 shows the results for ethane, propane, methane, and butene. The maximum yield of the key component, ethylene, occurs at 250 °C, followed by a decline at higher temperatures. Propylene and methane yield has similar characteristics. Toluene has a progressive rise in yield with increasing temperature, whereas ethane demonstrates an increase between 250 and 300 °C, followed by a marginal increase at 350 °C. 1-Butene exhibits a steady yield up to 290 °C, after which it abruptly increases at 300 °C, ultimately attaining a 7.92% yield at 350 °C. Benzene and isobutylene exhibit a rise in yield from 250 to 300 °C; however, for benzene, the yield reduces at 350 °C, while isobutylene yield remains mostly the same between 300 and 350 °C. Propane exhibits a distinctive trend compared to other compounds, with yield decreasing until 320 °C, followed by a rise in yield at 350 °C.

**Figure 5:**
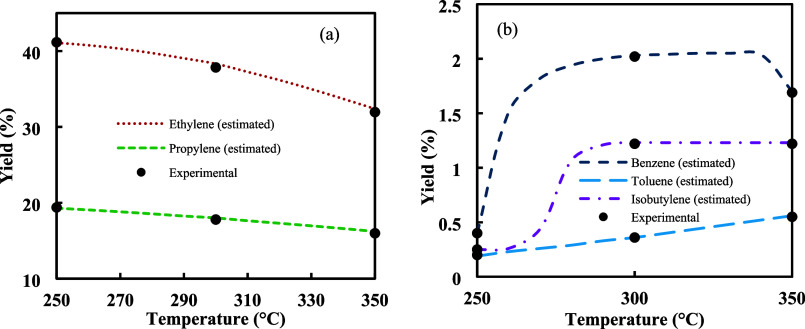
Estimated yield (%) variation with temperature between 250 and 350 °C for (a) ethylene and propylene, (b) benzene, toluene, and isobutylene.

**Figure 6:**
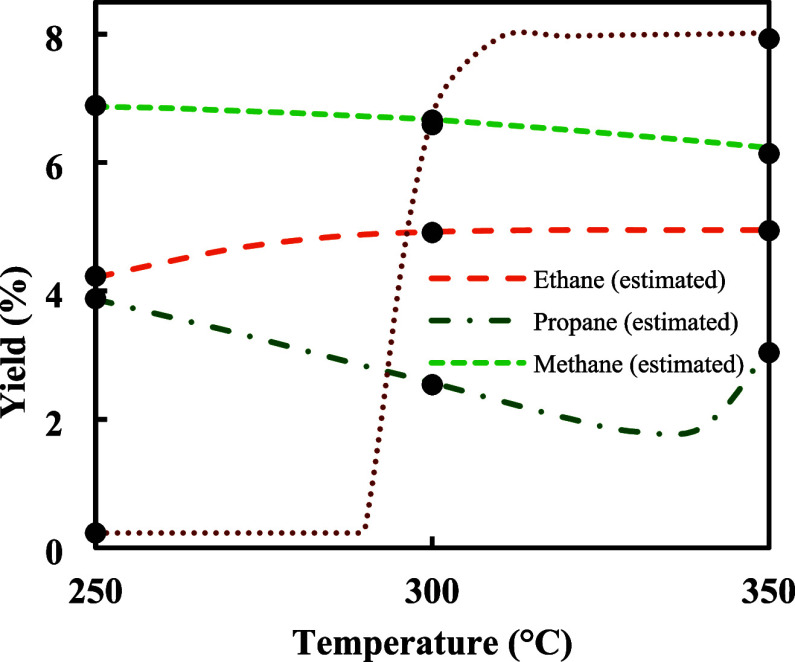
Estimated yield (%) variation with temperature between 250 and 350 °C for ethane, propane, methane, and butene.

As the ethylene yield is found to be the highest at 250 °C, it was desired to analyze how the ethylene production rate gets affected if the temperature is lowered below 250 °C. Furthermore, the highest yield (%) does not necessarily indicate that the highest production rate would be achieved at that temperature. This is because the reaction rate becomes slower at lower temperatures, so the reaction goes to completion over a longer batch time and therefore the total production rate (i.e., summation of product flow rates over time considering all products) would be lower. Thus, lower temperatures would lead to lower throughput per reactor, which is a measure of capital utilization. Figure 7 shows the average production rate of ethylene with respect to temperature for the laboratory-scale batch reactor by using the rate model and yield model that are extrapolated to simulate in the temperature range of 200–350 °C. It shows that the ethylene production rate (i.e., ethylene throughput from a plant incorporating the reactor) is not the highest at 250 °C, where the maximum ethylene yield is obtained, nor at 350 °C, where the highest reaction rate is obtained, but is around the intermediate temperature of 270 °C. It should be noted that commercial-scale production of ethylene by using this batch MW-assisted catalytic LDPE pyrolysis technology would not only require scale-up of this laboratory-scale reactor, but also several scaled-up reactors that are operated in parallel to achieve the desired throughput. Obviously, lower throughput from a single reactor would necessitate a larger number of reactors, thus adding to the capital cost.

**Figure 7:**
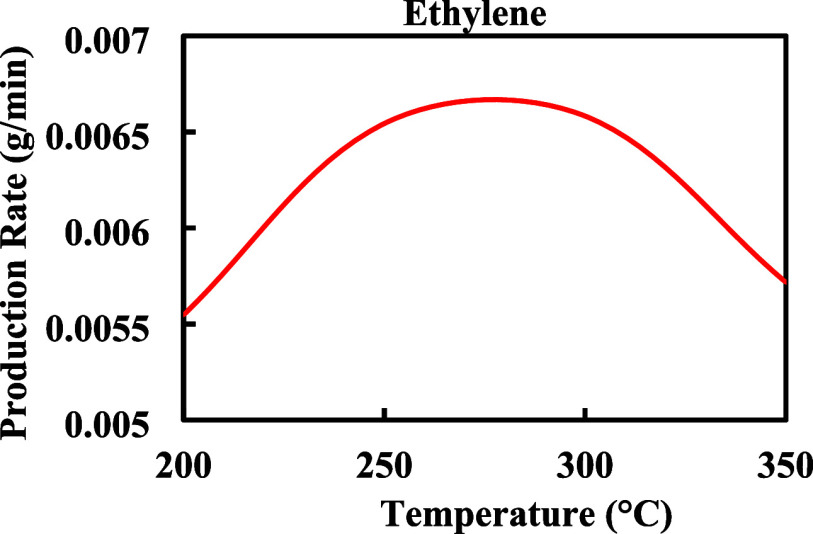
Sensitivity of the ethylene production rate to temperature.

## 5 Conclusions and Future Works

This work develops rate and yield models for MW-assisted catalytic pyrolysis with LDPE. The Arrhenius-type rate equation with a temperature-dependent reaction order is found to represent the experimental data well with an average RMSE value of 2.95%. It is observed that in the 250–350 °C temperature range that was studied experimentally, transient profiles were similar—cumulative conversion increases rapidly during the initial reaction period and gradually approaches complete consumption of LDPE.

A major contribution of this study is the development of a biexponential yield model coupled with sigmoidal temperature correlations, which was found to represent the complex, highly nonlinear characteristics of product yield with respect to the fraction of mass consumed and operating temperature very well. The RMSE for yield between the model results and the reconciled experimental data is found to be well below 10% for hydrogen, methane, ethylene, propylene, and propane. While the RMSE for benzene, toluene, and isobutylene yield is higher than 10%, production rates of these components are significantly lower than the key products of interest, i.e., ethylene and propylene; therefore, it is not likely to have much impact on the intended use of the developed models for process design and economic analysis. The kinetic and yield models are coupled to compute the product flow rates. The computed product flow rates are found to match the experimental data well with the average RMSE for ethylene and propylene flow rates being 8.6% and 9.65%, respectively.

The developed model is used to perform sensitivity analysis with respect to temperature. In particular, it was desired to find the optimal temperature for maximizing the average ethylene production rate from the batch reactor. It shows that the average ethylene production rate is around 270 °C, as opposed to 250 °C, where the maximum ethylene yield is obtained, or 350 °C, where the highest reaction rate is obtained. The model will be used in the future for scale-up, plant-wide process modeling, and techno-economic analysis.

Future work should investigate the effect of catalyst deactivation through dedicated experiments. Due to the short durations of the batch time in current experiments and as fresh catalysts are used in each experiment, catalyst deactivation is found to be negligible. However, if the catalyst is used over many batches, or reaction severity is enhanced, or the batch time is increased, then there may be considerable catalyst deactivation that can affect reactor performance. In the absence of any experimental observation, it is difficult to predict how catalyst deactivation would impact the reactor performance and how the model should be adapted to account for it. Nevertheless, it is anticipated that catalyst deactivation might affect both the reaction rate and yield and therefore can affect the activation energy, temperature dependence of reaction order, and/or yield models and their parameters. It is also desirable to undertake uncertainty quantification of the rate models and their parameters by considering uncertainty in the experimental data, especially for trace products such as toluene and isobutylene.

## Supplementary Material



## Notes

Additional results for data reconciliation, dynamic parameter estimation, yield model, and yield model coupled with the rate model are provided (PDF)
