# Supplementary material for: Microwave-Assisted Plastic Upcycling: Dynamic Data Reconciliation, Parameter Estimation, and Kinetic Modeling
Source: Ind Eng Chem Res. 2026 Jul 2;65(27):14461–71. doi: 10.1021/acs.iecr.6c01330 (PMC13383631; doi:10.1021/acs.iecr.6c01330)
Supplement: Supplementary file 1 [file ie6c01330_si_001.pdf]

Supplementary Information

Microwave-Assisted Plastic Upcycling: Dynamic Data Reconciliation, Parameter Estimation and Kinetic Modeling

Harish Damahe, Md Emdadul Haque, Chunlin Luo, Yuxin Wang, Jianli Hu, Debangsu Bhattacharyya*

Department of Chemical and Biomedical Engineering

West Virginia University, Morgantown, WV 26506, USA

*Corresponding author: Debangsu Bhattacharyya, Phone: 304-293-9355; E-mail: debangsu.bhattacharyya@mail.wvu.edu

Supplementary Information (SI)

The following items are provided in the supplementary information document for brevity:

- Figures S1 – S2
- Tables S1 – S4

Additional Results of Yield Model

Fig. S1 shows the yield model results for the remaining species, including hydrogen, methane, propane, propylene, isobutylene, and toluene.

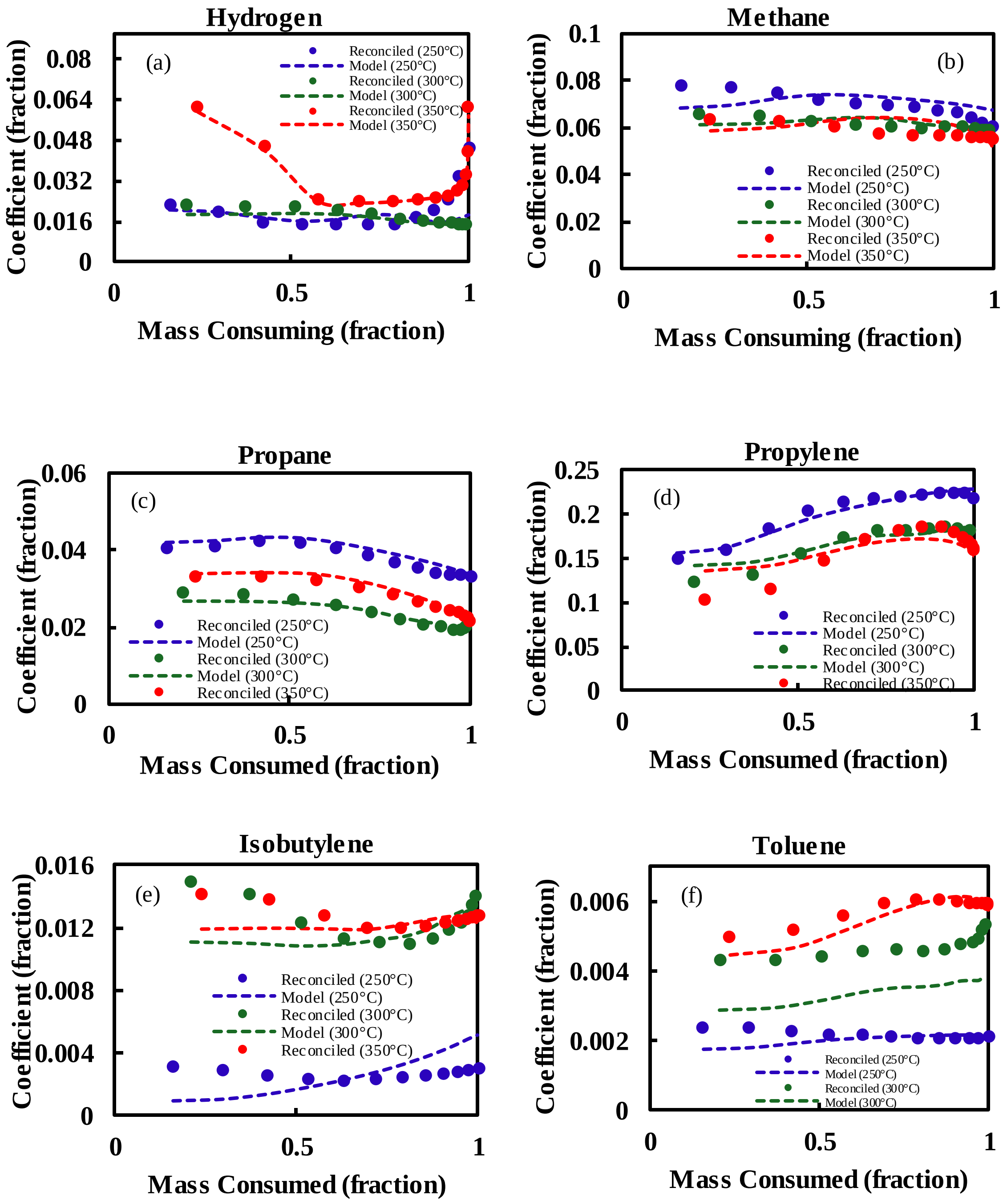

Fig. S1. Comparison between reconciled data and model results for yield of (a) hydrogen, (b) methane, (c) propane (d) propylene (e) isobutylene, and (f) toluene at 250°C, 300°C, and 350°C.

Additional Results on Comparison for the Product Flowrate

Fig. S2 shows the results of yield model coupled with rate model for the remaining species, including methane, hydrogen, propane, propylene, isobutylene, and toluene.

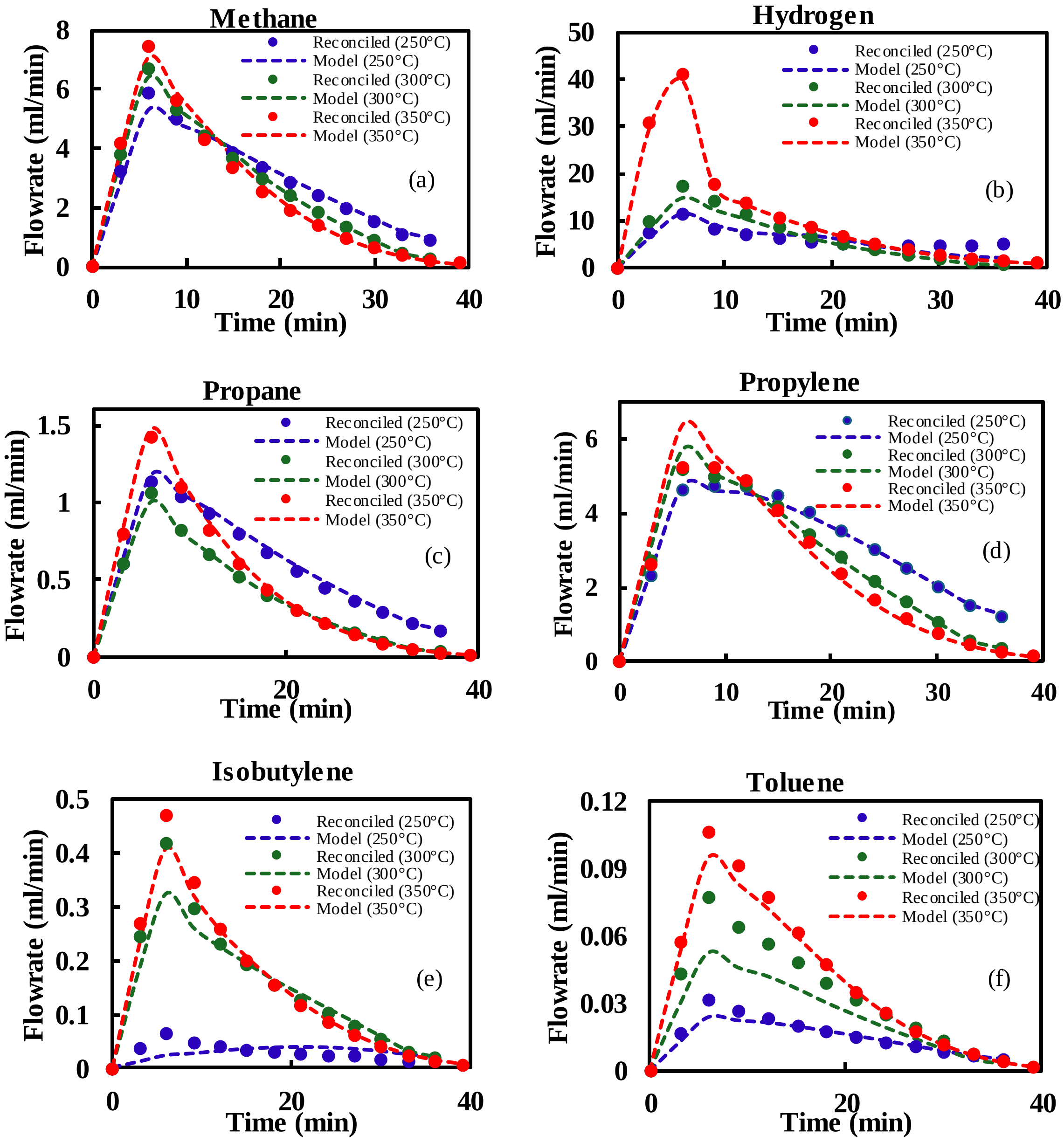

Fig. S2. Comparison between reconciled data and model results for flowrate of (a) methane (b) hydrogen (c) propane, (d) propylene, (e) isobutylene, and (f) toluene at 250°C, 300°C, and 350°C.

Table. S1 lists the estimated parameters corresponding to the yield model.

Table S1. Estimated Parameters for the Yield Model for the MW-assisted Pyrolysis of LDPE

Parameters	Methane	Ethylene	Ethane	Propane	Propylene	1-Butene	Toluene	Isobutylene	Carbon
a_1	4.0×10^{-4}	4.2×10^{-2}	4.9×10^{-2}	7.3×10^{-4}	1.4×10^{-1}	9.9×10^{-3}	4.0×10^{-4}	1.3×10^{-1}	0.24
b_1	0.98	1.6×10^{-2}	9.75	1.9×10^{-1}	8.4×10^{-3}	-899.25	1.99	-52.07	0.12
b_2	1.22	-2.00	-0.44	9.03	-2.00	0.86	0.21	-30.47	0.58
b_3	0.32	-0.48	0.49	-0.52	-0.48	0.23	-0.11	258.99	-0.02
c_1	0.52	0.26	20.04	-56.55	0.17	0.20	-90	99.99	233.10
c_2	0.15	3.29	-0.85	1.03	3.28	100.43	84	0.801	0.77
c_3	0.12	-0.87	11.69	1.36	-0.91	1.89	20.68	20.14	107.45
d_1	667.06	158.42	208.55	1920.17	158.53	15.75	-115.39	10877.61	196.55
e_1	659.35	157.00	-0.26	1917.74	157.08	15.18	-116.23	-1.00	-0.28
b_{sc}	11.47	390.35	1200.1	123.33	462.46	3.16	10.06	3.801	1193.49
c_{sc}	4.19	6.64	0.55	1.78	6.54	0.82	0.12	0.40	0.15

Table S2 summarizes the RMSE for the yield model and product flowrates calculated from the model vs. those from the reconciled experimental data. The values listed in Table S2 are the average values considering the entire batch time for all three temperatures.

Table S2. RMSE for the Yield Model and Product Flowrate.

Parameters	Methane	Ethylene	Ethane	Propane	Propylene	Benzene	Toluene	Isobutylene	Carbon
Yield Model	7.94	7.83	8.22	6.0	11.16	11.95	14.16	8.5**,28.25*	14.0
Flowrate	5.75	8.6	6.70	2.75	9.65	12.21	15.62	5.4**, 28.35*	1.27

** Excluding the 250°C data points, *Including all data points

Table. S3 summarizes the calculated average yield percentage of different species as a function of temperature indicating their temperature sensitivity. These values are calculated over the entire batch time.

Table S3. Calculated Yield Percentage for the MW-assisted Pyrolysis of LDPE

Temperature	Hydrogen Yield	Methane Yield	Ethylene Yield	Ethane Yield	Propane Yield	Propylene Yield	Butene Yield	Benzene Yield	Toluene Yield	Isobutylene Yield	Carbon Yield
250	2.32	6.77	41.96	4.18	3.86	18.6	0.25	0.4	0.19	0.23	21.31
260	2.19	6.86	41.72	4.49	3.67	18.75	0.25	1.47	0.23	0.25	20.17
270	2.31	6.84	40.82	4.7	3.43	18.52	0.25	1.8	0.26	0.42	20.71
280	2.33	6.85	40.06	4.89	3.19	18.38	0.25	1.96	0.29	1.05	20.8
290	2.46	6.84	39.21	5.03	2.94	18.21	0.25	2.05	0.33	1.23	21.51
300	1.86	6.75	37.87	5.04	2.66	17.82	6.67	2.07	0.36	1.24	17.72
310	1.79	6.76	37.04	5.16	2.41	17.73	8.14	2.13	0.4	1.26	17.24
320	2.01	6.72	35.92	5.2	2.14	17.49	8.22	2.16	0.44	1.27	18.49
330	2.25	6.67	34.72	5.23	1.93	17.23	8.3	2.18	0.49	1.28	19.78
340	2.45	6.61	33.44	5.27	2.01	16.96	8.38	2.17	0.53	1.29	20.96
350	2.96	6.05	32.3	4.91	3.07	15.57	7.84	1.67	0.54	1.21	23.95
250_Cal Exp	1.87	7.12	43.37	4.06	3.9	19.76	0.25	0.37	0.22	0.27	18.88
300_Cal Exp	2.63	6.18	36.45	4.55	2.48	15.99	6.43	2.01	0.46	1.25	21.64
350_Cal Exp	3.0	5.84	33.25	4.72	2.94	14.99	7.9	1.62	0.57	1.28	23.96

Table S4. Standard Deviation for the MW-assisted Pyrolysis of LDPE

Temperature	Hydrogen	Methane	Ethylene	Ethane	Propane	Propylene	Butene	Isobutylene	Benzene	Toluene
250	0.145	0.017	0.093	0.003	0.004	0.035	0.001	0.002	0.004	0.001
300	0.106	0.025	0.098	0.014	0.00	0.023	0.013	0.001	0.008	0.003
350	0.058	0.018	0.071	0.015	0.009	0.019	0.014	0.0009	0.012	0.005
